# Supplementary material for: γ-Protocadherin structural diversity and functional implications
Source: eLife. 2016 Oct 26;5:e20930. doi: 10.7554/eLife.20930 (PMC5106212; doi:10.7554/eLife.20930)
Supplement: Figure 1—source data 1. — Values in parentheses are for the outer shell. ASU = asymmetric unit; R.m.s. = Root mean square. DOI: http://dx.doi.org/10.7554/eLife.20930.004 [file elife-20930-fig1-data1.docx]

|  | **Pcdh γA1_EC1–4_** | **Pcdh γA8_EC1–4_** | **Pcdh γA9_EC1–5_** | **Pcdh γB2_EC1–5_** | **Pcdh γB7_EC1–4_**  **crystal form 1** | **Pcdh γB7_EC1–4_**  **crystal form 2** |
| --- | --- | --- | --- | --- | --- | --- |
| **Data collection** |  |  |  |  |  |  |
| Date | 03/24/2016 | 06/14/2014 | 06/26/2015 | 08/17/2016 | 10/25/2015 | 07/22/2016 |
| Beamline | APS 24ID-E | APS 24ID-E | APS 24ID-E | APS 24ID-E | APS 24ID-C | APS 24ID-C |
| Wavelength (Å) | 0.97918 | 0.97915 | 0.97918 | 0.97918 | 0.97930 | 0.97919 |
| Space group | P3_1_21 | *I*4_1_22 | *C*2 | *P*2_1_ | *P*4_1_2_1_2 | *P*2_1_ |
| *Cell dimensions* |  |  |  |  |  |  |
| a, b, c (Å) | 107.87, 107.87, 463.08 | 257.56, 257.56, 105.19 | 191.68, 107.61, 49.87 | 80.72, 118.07, 98.19 | 97.15, 97.15, 312.39 | 83.81, 45.55, 127.07 |
| α, β, γ (°) | 90, 90, 120 | 90, 90, 90 | 90, 97.14, 90 | 90, 103.91, 90 | 90, 90, 90 | 90, 96.91, 90 |
| Resolution (Å) | 40.00–4.20 (4.54–4.20) | 66.51−3.60 (3.97−3.60) | 38.80−2.94 (3.12−2.94) | 40.00–3.50 (3.78–3.50) | 104.13–3.59 (3.93–3.59) | 39.95–3.10 (3.31–3.10) |
| No. of reflections | 85269 | 146977 | 78312 | 85602 | 228986 | 59312 |
| Unique reflections | 23885 | 20729 | 21184 | 22663 | 18347 | 17677 |
| R_merge_ | 0.379 (2.646) | 0.172 (0.913) | 0.229 (3.156) | 0.369 (1.176) | 0.183 (3.722) | 0.180 (1.512) |
| CC(1/2) | 0.991 (0.318) | 0.998 (0.864) | 0.994 (0.582) | 0.957 (0.561) | 1.000 (0.741) | 0.982 (0.585) |
| I/σI | 3.1 (1.1) | 8.6 (2.3) | 4.5 (0.6) | 4.8 (1.6) | 9.0 (1.0) | 5.5 (0.8) |
| Completeness (%) | 99.9 (100.0) | 99.8 (100.0) | 98.9 (98.8) | 99.9 100.0) | 99.6 (98.7) | 99.5 (99.2) |
| Redundancy | 3.6 (3.6) | 7.1 (7.3) | 3.7 (3.8) | 3.8 (3.8) | 12.5 (12.3) | 3.4 (3.3) |
| **Refinement** |  |  |  |  |  |  |
| Resolution (Å) | 40–4.2 | 20–3.6 | 20–2.94/4.3/3.2 | 40–3.5 | 20–4.5/4.5/3.6 | 20–3.1 |
| Unique reflections | 23652 | 20598 | 13469 | 22631 | 11902 | 17214 |
| Completeness in diffracting sphere/ ellipsoid* (%) | 99.2 | 99.8 | 92.9* | 99.9 | 90.4* | 97.2 |
| R_work_ / R_free_ (%) | 28.66 / 31.36 | 21.09 / 23.78 | 24.11 / 29.54 | 21.31 / 25.65 | 24.21 / 27.87 | 25.58 / 30.98 |
| Molecules in ASU | 4 | 1 | 1 | 2 | 2 | 2 |
| *Number of residues* |  |  |  |  |  |  |
| Protein | 1659 | 416 | 523 | 1052 | 826 | 818 |
| Carbohydrate | 22 | 8 | 7 | 18 | 10 | 8 |
| Small molecule | 0 | 0 | 2 | 0 | 0 | 1 |
| Ion | 36 | 9 | 15 | 26 | 18 | 18 |
| Water | 0 | 0 | 37 | 4 | 0 | 5 |
| *R.m.s. deviations* |  |  |  |  |  |  |
| Bond lengths (Å) | 0.004 | 0.014 | 0.002 | 0.003 | 0.003 | 0.002 |
| Bond angles (°) | 0.822 | 0.564 | 0.667 | 0.613 | 0.588 | 0.585 |
| *Ramachandran* |  |  |  |  |  |  |
| Favored (%) | 94.55 | 95.89 | 94.61 | 96.18 | 95.26 | 96.56 |
| Allowed (%) | 5.45 | 3.86 | 5.39 | 3.82 | 4.74 | 3.44 |
| Outliers (%) | 0.00 | 0.24 | 0.00 | 0.00 | 0.00 | 0.00 |
| Rotamer outliers (%) | 0.31 | 0.00 | 0.71 | 0.00 | 1.78 | 0.00 |
| Wilson B | 133.85 | 104.64 | 53.84 | 69.73 | 109.88 | 108.83 |
| Overall B | 196.70 | 152.95 | 99.40 | 78.70 | 235.12 | 81.64 |
| Refined overall anisotropic scale matrix from Phenix | V0:  -5.7665, 19.8605, 6.3841, -1.7261, -1.4833, -8.2477  V1:  -0.0158, -0.1990, 0.0344, 0.0223, 0.0090, 0.0596 | V0:  -1.4787,  -12.6681, -5.2682, 0.2529, -1.0922, 6.8197  V1:  0.0201, 0.0764, -0.0236, 0.0030, 0.0048, -0.0296 | V0:  -1.4987,  -17.3398, 0.3982, -1.3034, -2.9210, 0.1157  V1:  0.0434, -0.0101, 0.0398, -0.0019, -0.0211, 0.0057 | V0:  0.4779, 2.9895,  -2.3112, -2.6096, 1.9457, 1.7383  V1:  -0.0210, 0.0508, -0.0043, 0.0170, 0.0222, -0.0129 | V0:  -7.4819,  -20.2922, 44.3031, 1.2023, -14.6157, 4.0158  V1:  -0.0390, -0.0504, -0.0239, -0.0238, 0.0455, 0.0117 | V0:  1.5887, -1.9187,  -1.5473, -1.1425, 2.7567, 0.0858  V1:  0.0375, -0.0130, -0.0095, 0.0202,  0.0323, -0.0105 |
| PDB ID | 5SZL | 5SZM | 5SZN | 5T9T | 5SZO | 5SZP |

#### Figure 1—source data 1. X-ray crystallography data collection and refinement statistics

Values in parentheses are for the outer shell. ASU = asymmetric unit; R.m.s. = Root mean square.
