# Supplementary material for: γ-Protocadherin structural diversity and functional implications
Source: eLife. 2016 Oct 26;5:e20930. doi: 10.7554/eLife.20930 (PMC5106212; doi:10.7554/eLife.20930)
Supplement: Figure 1—source data 2. — Root mean square deviations over aligned Cα’s (RMSDs) between pairs of individual Pcdh protomers. The number of aligned Cα’s for each pair is given in parentheses. xtal 1 = crystal form 1; xtal 2 = crystal form 2. The α4EC1–4, α7EC1–5, β6EC1–4, β8EC1–4, and γB3EC1–4 structures correspond to PDBs: 5DZW, 5DZV, 5DZX, 5DZY, and 5K8R. RMSDs between pairs of protomers from the same subfamily are shaded by subfamily. DOI: http://dx.doi.org/10.7554/eLife.20930.005 [file elife-20930-fig1-data2.docx]

| **Monomer RMSDs** | **α4_EC1­–4_** | **α7_EC1–5_ chain A** | **β6_EC1­–4_ chain A** | **β8_EC1­–4_ chain A** | **γA1_EC1­–4_ chain A** | **γA1_EC1­–4_ chain B** | **γA1_EC1­–4_ chain C** | **γA1_EC1­–4_ chain D** | **γA8_EC1­–4_** | **γA9_EC1­–5_** | **γB2_EC1­–5_**  **chain A** | **γB2_EC1­–5_**  **chain B** | **γB3_EC1­–4_** | **γB7_EC1­–4_ xtal 1 chain A** | **γB7_EC1­–4_ xtal 1 chain B** | **γB7_EC1­–4_ xtal 2 chain A** | **γB7_EC1­–4_ xtal 2 chain B** |
| --- | --- | --- | --- | --- | --- | --- | --- | --- | --- | --- | --- | --- | --- | --- | --- | --- | --- |
| **α4_EC1­–4_** |  | 1.5 Å (390) | 2.0 Å (364) | 2.0 Å (383) | 3.1 Å (398) | 3.1 Å (400) | 3.6 Å (404) | 5.5 Å (401) | 6.0 Å (407) | 5.7 Å (403) | 2.6 Å  (382) | 2.7 Å  (394) | 3.7 Å  (389) | 1.3 Å (384) | 1.9 Å (391) | 1.3 Å (361) | 1.5 Å (383) |
| **α7_EC1–5_ chain A** | 1.5 Å (390) |  | 2.0 Å (381) | 2.1 Å (382) | 3.4 Å  (402) | 3.3 Å (401) | 3.4 Å (411) | 5.1 Å (405) | 5.4 Å (404) | 5.5 Å (507) | 2.5 Å  (471) | 2.1 Å  (478) | 3.5 Å  (392) | 1.8 Å (372) | 2.2 Å (395) | 1.3 Å (345) | 1.9 Å (389) |
| **β6_EC1­–4_ chain A** | 2.0 Å (364) | 2.0 Å (381) |  | 1.7 Å (367) | 3.5 Å  (406) | 3.5 Å (400) | 3.6 Å (393) | 4.7 Å (375) | 4.9 Å (373) | 4.6 Å (366) | 3.0 Å  (393) | 3.0 Å  (394) | 3.0 Å  (372) | 2.4 Å (371) | 2.8 Å (394) | 2.1 Å (363) | 2.3 Å (384) |
| **β8_EC1­–4_ chain A** | 2.0 Å (383) | 2.1 Å (382) | 1.7 Å (367) |  | 3.4 Å  (404) | 3.0 Å (399) | 3.7 Å (407) | 5.3 Å (404) | 4.9 Å (390) | 4.9 Å (390) | 2.3 Å  (389) | 2.5 Å  (392) | 2.5 Å  (391) | 2.8 Å (398) | 3.1 Å (394) | 2.2 Å (388) | 2.7 Å (389) |
| **γA1_EC1­–4_ chain A** | 3.1 Å (398) | 3.4 Å  (402) | 3.5 Å  (406) | 3.4 Å  (404) |  | 0.9 Å (389) | 2.9 Å (398) | 4.9 Å (402) | 5.0 Å (372) | 5.8 Å (390) | 4.5 Å  (395) | 4.5 Å  (394) | 4.8 Å  (392) | 3.1 Å (401) | 3.3 Å (400) | 2.8 Å (397) | 3.4 Å (400) |
| **γA1_EC1­–4_ chain B** | 3.1 Å (400) | 3.3 Å (401) | 3.5 Å (400) | 3.0 Å (399) | 0.9 Å (389) |  | 2.4 Å (375) | 3.2 Å (348) | 3.9 Å (348) | 5.8 Å (400) | 4.2 Å  (397) | 4.3 Å  (398) | 4.6 Å  (406) | 3.2 Å (398) | 3.4 Å (400) | 2.7 Å (395) | 2.7 Å (389) |
| **γA1_EC1­–4_ chain C** | 3.6 Å (404) | 3.4 Å (411) | 3.6 Å (393) | 3.7 Å (407) | 2.9 Å (398) | 2.4 Å (375) |  | 1.9 Å (404) | 2.0 Å (335) | 3.6 Å (398) | 3.9 Å  (396) | 3.7 Å  (395) | 4.6 Å  (410) | 3.8 Å (407) | 3.1 Å (405) | 2.6 Å (392) | 3.6 Å (403) |
| **γA1_EC1­–4_ chain D** | 5.5 Å (401) | 5.1 Å (405) | 4.7 Å (375) | 5.3 Å (404) | 4.9 Å (402) | 3.2 Å (348) | 1.9 Å (404) |  | 1.2 Å (336) | 2.0 Å (345) | 4.8 Å (382) | 4.6 Å  (384) | 5.2 Å  (399) | 6.0 Å (411) | 5.1 Å (407) | 4.6 Å (402) | 5.4 Å (400) |
| **γA8_EC1­–4_** | 6.0 Å (407) | 5.4 Å (404) | 4.9 Å (373) | 5.6 Å (387) | 5.0 Å (372) | 3.9 Å (348) | 2.0 Å (335) | 1.2 Å (336) |  | 3.3 Å  (398) | 5.3 Å  (378) | 4.9 Å  (380) | 5.2 Å  (373) | 6.2 Å (408) | 5.0 Å (401) | 5.0 Å (403) | 5.4 Å (396 |
| **γA9_EC1­–5_** | 5.7 Å (403) | 5.5 Å (507) | 4.6 Å (366) | 4.9 Å (390) | 5.8 Å (390) | 5.8 Å (400) | 3.6 Å (398) | 2.0 Å (345) | 3.3 Å  (398) |  | 5.5 Å  (516) | 5.5 Å  (518) | 3.9 Å  (394) | 6.0 Å (391) | 5.3 Å (391) | 4.8 Å (388) | 5.4 Å (388) |
| **γB2_EC1­–5_**  **chain A** | 2.6 Å  (382) | 2.5 Å  (471) | 3.0 Å  (393) | 2.3 Å  (389) | 4.5 Å  (395) | 4.2 Å  (397) | 3.9 Å  (396) | 4.8 Å (382) | 5.3 Å  (378) | 5.5 Å  (516) |  | 2.3 Å  (500) | 2.2 Å  (403) | 3.1 Å  (398) | 3.6 Å  (410) | 3.0 Å  (390) | 2.7 Å  (395) |
| **γB2_EC1­–5_**  **chain B** | 2.7 Å  (394) | 2.1 Å  (478) | 3.0 Å  (394) | 2.5 Å  (392) | 4.5 Å  (394) | 4.3 Å  (398) | 3.7 Å  (395) | 4.6 Å  (384) | 4.9 Å  (380) | 5.5 Å  (518) | 2.3 Å  (500) |  | 2.4 Å  (395) | 2.9 Å  (386) | 3.2 Å  (397) | 2.9 Å  (389) | 2.6 Å  (396) |
| **γB3_EC1­–4_** | 3.7 Å  (389) | 3.5 Å  (392) | 3.0 Å  (372) | 2.5 Å  (391) | 4.8 Å  (392) | 4.6 Å  (406) | 4.6 Å  (410) | 5.2 Å  (399) | 5.2 Å  (373) | 3.9 Å  (394) | 2.2 Å  (403) | 2.4 Å  (395) |  | 4.7 Å  (409) | 4.1 Å  (372) | 3.9 Å  (400) | 3.7 Å  (372) |
| **γB7_EC1­–4_**  **xtal 1 chA** | 1.3 Å (384) | 1.8 Å (372) | 2.4 Å (371) | 2.8 Å (398) | 3.1 Å (401) | 3.2 Å (398) | 3.8 Å (407) | 6.0 Å (411) | 6.2 Å (408) | 6.0 Å (391) | 3.1 Å  (398) | 2.9 Å  (386) | 4.7 Å  (409) |  | 1.3 Å (398) | 1.5 Å (408) | 1.2 Å (396) |
| **γB7_EC1­–4_**  **xtal 1 chB** | 1.9 Å (391) | 2.2 Å (395) | 2.8 Å (394) | 3.1 Å (394) | 3.3 Å (400) | 3.4 Å (400) | 3.1 Å (405) | 5.1 Å (407) | 5.0 Å (401) | 5.3 Å (391) | 3.6 Å  (410) | 3.2 Å  (397) | 4.1 Å  (372) | 1.3 Å (398) |  | 1.4 Å (398) | 1.0 Å (380) |
| **γB7_EC1­–4_**  **xtal 2 chA** | 1.3 Å (361) | 1.3 Å (345) | 2.1 Å (363) | 2.2 Å (388) | 2.8 Å (397) | 2.7 Å (395) | 2.6 Å (392) | 4.6 Å (402) | 5.0 Å (403) | 4.8 Å (388) | 3.0 Å  (390) | 2.9 Å  (389) | 3.9 Å  (400) | 1.5 Å (408) | 1.4 Å (398) |  | 1.4 Å (383) |
| **γB7_EC1­–4_**  **xtal 2 chB** | 1.5 Å (383) | 1.9 Å (389) | 2.3 Å (384) | 2.7 Å (389) | 3.4 Å (400) | 2.7 Å (389) | 3.6 Å (403) | 5.4 Å (400) | 5.4 Å (396) | 5.4 Å (388) | 2.7 Å  (395) | 2.6 Å  (396) | 3.7 Å  (372) | 1.2 Å (396) | 1.0 Å (380) | 1.4 Å (383) |  |

#### Figure 1—source data 2. Overall structural similarity between EC1–4 regions of α-, β-, and γ-Pcdh structures

Root mean square deviations over aligned Cα’s (RMSDs) between pairs of Pcdh individual protomers. The number of aligned Cα’s for each pair is given in parentheses. xtal 1 = crystal form 1; xtal 2 = crystal form 2. The α4_EC1–4_, α7_EC1–5_, β6_EC1–4_, β8_EC1–4_, and γB3_EC1–4_ structures correspond to PDBs: 5DZW, 5DZV, 5DZX, 5DZY, and 5K8R. RMSDs between pairs of protomers from the same subfamily are shaded by subfamily.
