# Supplementary material for: γ-Protocadherin structural diversity and functional implications
Source: eLife. 2016 Oct 26;5:e20930. doi: 10.7554/eLife.20930 (PMC5106212; doi:10.7554/eLife.20930)
Supplement: Figure 1—source data 4. — Root mean square deviations over aligned Cα’s (RMSDs) between pairs of interacting EC2–3:EC2–3 regions from the Pcdh trans dimer structures are shown. The number of aligned Cα’s for each pair is given in parentheses. The α4EC1–4, α7EC1–5, β6EC1–4, β8EC1–4, and γB3EC1–4 structures correspond to PDBs: 5DZW, 5DZV, 5DZX, 5DZY, and 5K8R. RMSDs between pairs of dimers from the same subfamily are shaded by subfamily. DOI: http://dx.doi.org/10.7554/eLife.20930.007 [file elife-20930-fig1-data4.docx]

| **EC23 RMSDs** | **α4** | **α7** | **β6** | **β8**  **(chains A&B)** | **γA1**  **(chains A&B)** | **γA1**  **(chains C&D)** | **γA8** | **γB2** | **γB3** | **γB7**  **(crystal form 1)** | **γB7**  **(crystal form 2)** |
| --- | --- | --- | --- | --- | --- | --- | --- | --- | --- | --- | --- |
| **α4** |  | 1.3 Å  (389) | 4.7 Å  (391) | 3.9 Å  (385) | 4.4 Å (389) | 4.6 Å (386) | 6.2 Å (393) | 2.1 Å  (390) | 4.1 Å  (393) | 1.6 Å (377) | 1.4 Å (356) |
| **α7** | 1.3 Å  (389) |  | 4.7 Å  (398) | 4.0 Å  (391) | 4.5 Å (408) | 4.7 Å (404) | 6.1 Å (406) | 2.1 Å  (380) | 4.0 Å  (401) | 2.2 Å (384) | 2.0 Å (370) |
| **β6** | 4.7 Å  (391) | 4.7 Å  (398) |  | 1.0 Å  (398) | 3.2 Å (393) | 2.9 Å (371) | 3.6 Å (368) | 4.1 Å  (406) | 1.9 Å  (392) | 4.1 Å (401) | 3.9 Å (398) |
| **β8**  **(chains A&B)** | 3.9 Å  (385) | 4.0 Å  (391) | 1.0 Å  (398) |  | 3.3 Å (395) | 2.9 Å (370) | 3.9 Å (369) | 3.5 Å  (405) | 1.9 Å  (395) | 3.4 Å (398) | 3.3 Å (397) |
| **γA1**  **(chains A&B)** | 4.4 Å (389) | 4.5 Å (408) | 3.2 Å (393) | 3.3 Å (395) |  | 0.8 Å (385) | 2.9 Å (407) | 4.4 Å  (402) | 3.4 Å  (408) | 4.1 Å (381) | 4.0 Å (380) |
| **γA1**  **(chains C&D)** | 4.6 Å (386) | 4.7 Å (404) | 2.9 Å (371) | 2.9 Å (370) | 0.8 Å (385) |  | 2.3 Å (403) | 4.5 Å  (398)) | 3.1 Å  (394) | 3.9 Å (358) | 3.8 Å (363) |
| **γA8** | 6.2 Å (393) | 6.1 Å (406) | 3.6 Å (368) | 3.9 Å (369) | 2.9 Å (407) | 2.3 Å (403) |  | 5.9 Å  (405) | 3.3 Å  (373) | 5.3 Å (367) | 5.3 Å (374) |
| **γB2** | 2.1 Å  (390) | 2.1 Å  (380) | 4.1 Å  (406) | 3.5 Å  (405) | 4.4 Å  (402) | 4.5 Å  (398) | 5.9 Å  (405) |  | 3.7 Å  (408) | 1.8 Å  (399) | 1.6 Å  (393) |
| **γB3** | 4.1 Å  (393) | 4.0 Å  (401) | 1.9 Å  (392) | 1.9 Å  (395) | 3.4 Å  (408) | 3.1 Å  (394) | 3.3 Å  (373) | 3.7 Å  (408) |  | 4.0 Å  (404) | 3.9 Å  (404) |
| **γB7**  **(crystal form 1)** | 1.6 Å (377) | 2.2 Å (384) | 4.1 Å (401) | 3.4 Å (398) | 4.1 Å (381) | 3.9 Å (358) | 5.3 Å (367) | 1.8 Å  (399) | 4.0 Å  (404) |  | 0.8 Å (391) |
| **γB7**  **(crystal form 2)** | 1.4 Å (356) | 2.0 Å (370) | 3.9 Å (398) | 3.3 Å (397) | 4.0 Å (380) | 3.8 Å (363) | 5.3 Å (374) | 1.6 Å  (393) | 3.9 Å  (404) | 0.8 Å (391) |  |

#### Figure 1—source data 4. Overall structural similarity between α-, β-, and γ-Pcdh EC2:EC3 interface regions

Root mean square deviations over aligned Cα’s (RMSDs) between pairs of interacting EC2–3:EC2–3 regions from the Pcdh *trans* dimer structures are shown. The number of aligned Cα’s for each pair is given in parentheses. The α4_EC1–4_, α7_EC1–5_, β6_EC1–4_, β8_EC1–4_, and γB3_EC1–4_ structures correspond to PDBs: 5DZW, 5DZV, 5DZX, 5DZY, and 5K8R. RMSDs between pairs of dimers from the same subfamily are shaded by subfamily.
