# Supplementary material for: γ-Protocadherin structural diversity and functional implications
Source: eLife. 2016 Oct 26;5:e20930. doi: 10.7554/eLife.20930 (PMC5106212; doi:10.7554/eLife.20930)
Supplement: Figure 1—source data 6. — Interdomain angles between consecutive EC domains given as the deviation from 180°, were calculated using UCSF Chimera. The α4EC1–4, α7EC1–5, β6EC1–4, β8EC1–4, and γB3EC1–4 structures correspond to PDBs: 5DZW, 5DZV, 5DZX, 5DZY, and 5K8R. DOI: http://dx.doi.org/10.7554/eLife.20930.009 [file elife-20930-fig1-data6.docx]

| **Interdomain angles** | **EC1–EC2 (°)** | **EC2–EC3 (°)** | **EC3–EC4 (°)** | **EC4–EC5 (°)** |
| --- | --- | --- | --- | --- |
| **α4_EC1­–4_** | 12.0 | 7.0 | 14.0 |  |
| **α7_EC1–5_ chain A** | 14.9 | 6.3 | 11.4 | 21.9 |
| **α7_EC1–5_ chain B** | 14.2 | 8.4 | 11.5 | 22.5 |
| ***Average α-Pcdh*** | *13.7 ± 1.5* | *7.2 ± 1.1* | *12.3 ± 1.5* | *22.2 ± 0.4* |
| **β6_EC1­–4_ chain A** | 13.3 | 11.0 | 8.6 |  |
| **β6_EC1­–4_ chain B** | 13.5 | 10.4 | 6.3 |  |
| **β8_EC1­–4_ chain A** | 6.0 | 10.1 | 18.2 |  |
| **β8_EC1­–4_ chain B** | 5.0 | 9.1 | 10.4 |  |
| **β8_EC1­–4_ chain C** | 4.7 | 7.1 | 14.9 |  |
| **β8_EC1­–4_ chain D** | 2.3 | 8.8 | 15.1 |  |
| **β8_EC1­–4_ chain E** | 7.0 | 11.6 | 15.4 |  |
| **β8_EC1­–4_ chain F** | 4.6 | 13.9 | 13.5 |  |
| ***Average β-Pcdh*** | *7.1 ± 4.1* | *10.3 ± 2.0* | *12.8 ± 4.0* |  |
| **γA1_EC1­–4_ chain A** | 12.3 | 18.8 | 24.0 |  |
| **γA1_EC1­–4_ chain B** | 4.9 | 17.0 | 25.0 |  |
| ***Average EC1–4 engaged γA-Pcdh*** | *8.6 ± 5.2* | *17.9 ± 1.3* | *24.5 ± 0.7* |  |
| **γA1_EC1­–4_ chain C** | 8.4 | 21.6 | 16.8 |  |
| **γA1_EC1­–4_ chain D** | 5.8 | 22.3 | 7.9 |  |
| **γA8_EC1­–4_** | 7.3 | 26.3 | 9.2 |  |
| **γA9_EC1­–5_** | 10.8 | 21.9 | 8.1 | 17.7 |
| ***Average not fully engaged γA-Pcdh*** | *8.1 ± 2.1* | *23.0 ± 2.2* | *10.5 ± 4.2* |  |
| **γB2_EC1­–5_ chain A** | 21.2 | 12.2 | 17.2 | 15.4 |
| **γB2_EC1­–5_ chain B** | 22.5 | 11.7 | 17.1 | 16.9 |
| **γB3_EC1­–4_** | 9.7 | 13.3 | 17.5 |  |
| **γB7_EC1­–4_ xtal 1 chain A** | 12.0 | 6.1 | 15.2 |  |
| **γB7_EC1­–4_ xtal 1 chain B** | 11.4 | 10.0 | 15.7 |  |
| **γB7_EC1­–4_ xtal 2 chain A** | 4.8 | 6.0 | 10.2 |  |
| **γB7_EC1­–4_ xtal 2 chain B** | 14.4 | 8.4 | 16.1 |  |
| ***Average γB-Pcdh*** | *13.7 ± 6.3* | *9.7 ± 2.9* | *15.6 ± 2.5* | *16.2 ± 1.1* |

#### Figure 1—source data 6. Pcdh protomer interdomain angles

Interdomain angles between consecutive EC domains given as the deviation from 180°, were calculated using UCSF chimera. The α4_EC1–4_, α7_EC1–5_, β6_EC1–4_, β8_EC1–4_, and γB3_EC1–4_ structures correspond to PDBs: 5DZW, 5DZV, 5DZX, 5DZY, and 5K8R.
