# Supplementary material for: γ-Protocadherin structural diversity and functional implications
Source: eLife. 2016 Oct 26;5:e20930. doi: 10.7554/eLife.20930 (PMC5106212; doi:10.7554/eLife.20930)
Supplement: Figure 1—source data 7. — Interfacial buried surface areas (BSAs) are given as the difference in accessible surface area over both protomers upon dimer formation. BSAs were determined using the PISA server. Unmodeled side chains in the crystal structures were generated using the Dunbrack rotamer library in UCSF Chimera. The α4EC1–4, α7EC1–5, β6EC1–4, β8EC1–4, and γB3EC1–4 structures correspond to PDBs: 5DZW, 5DZV, 5DZX, 5DZY, and 5K8R. DOI: http://dx.doi.org/10.7554/eLife.20930.010 [file elife-20930-fig1-data7.docx]

| **Buried surface area (Å^2^)** | **α4** | **α7** | **β6** | **β8**  **(chains A&B)** | **γA1**  **(chains A&B)** | **γA1**  **(chains C&D)** | **γA8** | **γB2** | **γB3** | **γB7**  **(crystal form 1)** | **γB7**  **(crystal form 2)** |
| --- | --- | --- | --- | --- | --- | --- | --- | --- | --- | --- | --- |
| **Entire interface in crystal structure** | 4319 | 3316 | 4554 | 4821 | 3237 | 2641 | 1598 | 4006 | 3069 | 4190 | 3747 |
| **Entire interface including all side chains** | 4995 | 3904 | 4678 | 5093 | 3522 | 2703 | 1658 | 4275 | 3069 | 4601 | 4456 |
| **EC2:EC3 interface including all side chains** | 2922 | 1975 | 2476 | 2971 | 2527 | 2703 | 1658 | 1735 | 1114 | 2546 | 2846 |
| **EC1:EC4 interfaces including all side chains** | 1948 | 1929 | 2202 | 2102 | 997 | 0 | 0 | 2540 | 1904 | 1987 | 1621 |

#### Figure 1—source data 7. *Trans*-dimer buried surface areas in all Pcdh EC1–4 containing crystal structures

Interfacial buried surface areas (BSAs) are given as the difference in accessible surface area over both protomers upon dimer formation. BSAs were determined using the PISA server. Unmodeled side chains in the crystal structures were generated using the Dunbrack rotamer library in UCSF chimera. The α4_EC1–4_, α7_EC1–5_, β6_EC1–4_, β8_EC1–4_, and γB3_EC1–4_ structures correspond to PDBs: 5DZW, 5DZV, 5DZX, 5DZY, and 5K8R.
