# Supplementary material for: γ-Protocadherin structural diversity and functional implications
Source: eLife. 2016 Oct 26;5:e20930. doi: 10.7554/eLife.20930 (PMC5106212; doi:10.7554/eLife.20930)
Supplement: Figure 3—source data 1. — DOI: http://dx.doi.org/10.7554/eLife.20930.018 [file elife-20930-fig3-data1.docx]

| **Isoform** | **Number of orthologs** | **Species** |
| --- | --- | --- |
| γB1 | 13 | Mus musculus, Oryctolagus cuniculus, Rhinopithecus roxellana, Mandrillus leucophaeus, Macaca mulatta, Macaca fascicularis, Pongo abelii, Pan troglodytes, Pan paniscus, Gorilla gorilla gorilla, Physeter catodon, Camelus dromedarius, Leptonychotes weddellii |
| γB2 | 9 | Mus musculus, Monodelphis domestica, Chrysochloris asiatica, Fukomys damarensis, Bison bison bison, Leptonychotes weddellii, Rhinopithecus roxellana, Mandrillus leucophaeus, Pongo abelii |
| γB3 | 20 | Mus musculus, Oryctolagus cuniculus, Echinops telfairi, Gorilla gorilla gorilla, Pongo abelii, Rhinopithecus roxellana, Chlorocebus sabaeus, Mandrillus leucophaeus, Papio anubis, Macaca fascicularis, Macaca mulatta, Aotus nancymaae, Callithrix jacchus, Leptonychotes weddellii, Chrysochloris asiatica, Trichechus manatus latirostris, Camelus dromedarius, Physeter catodon, Tursiops truncatus, Lipotes vexillifer |
| γB4 | 14 | Mus musculus, Chrysochloris asiatica, Fukomys damarensis, Callithrix jacchus, Mandrillus leucophaeus, Pongo abelii, Nomascus leucogenys, Homo sapiens, Gorilla gorilla gorilla, Camelus dromedarius, Bubalus bubalis, Physeter catodon, Lipotes vexillifer, Tursiops truncatus |
| γB5 | 11 | Mus musculus, Fukomys damarensis, Chrysochloris asiatica, Mandrillus leucophaeus, Pongo abelii, Gorilla gorilla gorilla, Homo sapiens, Lipotes vexillifer, Physeter catodon, Pantholops hodgsonii, Bison bison bison |
| γB6 | 13 | Mus musculus, Fukomys damarensis, Pongo abelii, Colobus angolensis palliatus, Macaca nemestrina, Nomascus leucogenys, Gorilla gorilla gorilla, Homo sapiens, Bubalus bubalis, Pantholops hodgsonii, Lipotes vexillifer, Orcinus orca, Tursiops truncatus |
| γB7 | 20 | Mus musculus, Mesocricetus auratus, Peromyscus maniculatus bairdii, Octodon degus, Tupaia chinensis, Orcinus orca, Microcebus murinus, Bubalus bubalis, Pantholops hodgsonii, Lipotes vexillifer, Tursiops truncatus, Balaenoptera acutorostrata scammoni, Galeopterus variegatus, Colobus angolensis palliatus, Pongo abelii, Homo sapiens, Gorilla gorilla gorilla, Pan paniscus, Elephantulus edwardii, Orycteropus afer afer |
| γB8 | 30 | Mus musculus, Rattus norvegicus, Neotoma lepida, Peromyscus maniculatus bairdii, Cricetulus griseus, Microtus ochrogaster, Trichechus manatus latirostris, Rousettus aegyptiacus, Equus asinus, Pteropus alecto, Galeopterus variegatus, Tupaia chinensis, Heterocephalus glaber, Condylura cristata, Vicugna pacos, Ictidomys tridecemlineatus, Elephantulus edwardii, Felis catus, Carlito syrichta, Leptonychotes weddellii, Bubalus bubalis, Orycteropus afer afer, Cavia porcellus, Ursus maritimus, Propithecus coquereli, Saimiri boliviensis boliviensis, Odobenus rosmarus divergens, Echinops telfairi, Dasypus novemcinctus, Chinchilla lanigera |

#### Figure 3—source data 1. List of species used in generating the sequence logos for γB-Pcdh isoforms.

|  |  |  |
| --- | --- | --- |
|  |  |  |
|  |  |  |
|  |  |  |
