# Supplementary material for: γ-Protocadherin structural diversity and functional implications
Source: eLife. 2016 Oct 26;5:e20930. doi: 10.7554/eLife.20930 (PMC5106212; doi:10.7554/eLife.20930)
Supplement: Figure 4—source data 1. — DOI: http://dx.doi.org/10.7554/eLife.20930.022 [file elife-20930-fig4-data1.docx]

| **Isoform** | **Number of orthologs** | **Species** |
| --- | --- | --- |
| γA1 | 24 | Mus musculus, Rattus norvegicus, Dipodomys ordii, Jaculus jaculus, Heterocephalus glaber, Cavia porcellus, Octodon degus, Oryctolagus cuniculus, Elephantulus edwardii, Loxodonta africana, Echinops telfairi, Chrysochloris asiatica, Tarsius syrichta, Gorilla gorilla gorilla, Homo sapiens, Pan troglodytes, Pan paniscus, Colobus angolensis palliatus, Macaca nemestrina, Leptonychotes weddellii, Bubalus bubalis, Physeter catodon, Lipotes vexillifer, Tursiops truncatus |
| γA2 | 17 | Jaculus jaculus, Mus musculus, Rattus norvegicus, Fukomys damarensis, Octodon degus, Chrysochloris asiatica, Leptonychotes weddellii, Bubalus bubalis, Physeter catodon, Lipotes vexillifer, Orcinus orca, Tursiops truncatus, Galeopterus variegatus, Callithrix jacchus, Gorilla gorilla gorilla, Pan paniscus, Pan troglodytes, |
| γA3 | 17 | Chrysochloris asiatica, Echinops telfairi, Elephantulus edwardii, Homo sapiens, Pan troglodytes, Octodon degus, Fukomys damarensis, Galeopterus variegatus, Odobenus rosmarus divergens, Bubalus bubalis, Physeter catodon, Lipotes vexillifer, Orcinus orca, Tursiops truncatus, Jaculus jaculus, Mus musculus, Rattus norvegicus |
| γA4 | 15 | Fukomys damarensis, Octodon degus, Mus musculus, Jaculus jaculus, Dipodomys ordii, Sorex araneus, Rhinopithecus roxellana, Homo sapiens, Gorilla gorilla gorilla, Pan troglodytes, Tarsius syrichta, Leptonychotes weddellii, Odobenus rosmarus divergens, Physeter catodon, Bubalus bubalis |
| γA5 | 22 | Ochotona princeps, Trichechus manatus latirostris, Chrysochloris asiatica, Sorex araneus, Tarsius syrichta, Leptonychotes weddellii, Odobenus rosmarus divergens, Camelus dromedarius, Physeter catodon, Lipotes vexillifer, Orcinus orca, Callithrix jacchus, Colobus angolensis palliatus, Pan troglodytes, Gorilla gorilla gorilla, Homo sapiens, Otolemur garnettii, Propithecus coquereli, Fukomys damarensis, Octodon degus, Jaculus jaculus, Mus musculus |
| γA6 | 16 | Sorex araneus, Dipodomys ordii, Mus musculus, Cricetulus griseus, Nannospalax galili, Jaculus jaculus, Fukomys damarensis, Octodon degus, Leptonychotes weddellii, Odobenus rosmarus divergens, Tursiops truncatus, Lipotes vexillifer, Physeter catodon, Tarsius syrichta, Pan troglodytes, Gorilla gorilla gorilla |
| γA7 | 15 | Jaculus jaculus, Mus musculus, Cricetulus griseus, Octodon degus, Echinops telfairi, Tarsius syrichta, Pan troglodytes, Pan paniscus, Nomascus leucogenys, Sorex araneus, Leptonychotes weddellii, Odobenus rosmarus divergens, Bubalus bubalis, Lipotes vexillifer, Physeter catodon |
| γA8 | 15 | Elephantulus edwardii, Chrysochloris asiatica, Homo sapiens, Gorilla gorilla gorilla, Leptonychotes weddellii, Camelus dromedarius, Physeter catodon, Lipotes vexillifer, Tursiops truncatus, Octodon degus, Jaculus jaculus, Dipodomys ordii, Nannospalax galili, Mus musculus, Cricetulus griseus |
| γA9 | 13 | Leptonychotes weddellii, Elephantulus edwardii, Octodon degus, Jaculus jaculus, Mus musculus, Cricetulus griseus, Orycteropus afer afer, Sorex araneus, Bison bison bison, Bubalus bubalis, Pantholops hodgsonii, Homo sapiens, Trichechus manatus latirostris |
| γA10 | 16 | Monodelphis domestica, Mus musculus, Elephantulus edwardii, Jaculus jaculus, Chrysochloris asiatica, Sorex araneus, Octodon degus, Ictidomys tridecemlineatus, Propithecus coquereli, Colobus angolensis palliatus, Gorilla gorilla gorilla, Leptonychotes weddellii, Odobenus rosmarus divergens, Lipotes vexillifer, Bubalus bubalis, Pantholops hodgsonii |
| γA11 | 20 | Ochotona princeps, Echinops telfairi, Chrysochloris asiatica, Mus musculus, Jaculus jaculus, Octodon degus, Ictidomys tridecemlineatus, Galeopterus variegatus, Leptonychotes weddellii, Aotus nancymaae, Colobus angolensis palliatus, Rhinopithecus roxellana, Gorilla gorilla gorilla, Homo sapiens, Pan troglodytes, Bubalus bubalis, Balaenoptera acutorostrata scammoni, Lipotes vexillifer, Orcinus orca, Tursiops truncatus |
| γA12 | 19 | Chelonia mydas, Monodelphis domestica, Ochotona princeps, Mus musculus, Ictidomys tridecemlineatus, Jaculus jaculus, Nannospalax galili, Chrysochloris asiatica, Elephantulus edwardii, Octodon degus, Nomascus leucogenys, Gorilla gorilla gorilla, Cercocebus atys, Colobus angolensis palliatus, Eptesicus fuscus, Leptonychotes weddellii, Odobenus rosmarus divergens, Bubalus bubalis, Lipotes vexillifer |

#### Figure 4—source data 1. List of species used in generating the sequence logos for γA-Pcdh isoforms.
