# Supplementary material for: γ-Protocadherin structural diversity and functional implications
Source: eLife. 2016 Oct 26;5:e20930. doi: 10.7554/eLife.20930 (PMC5106212; doi:10.7554/eLife.20930)
Supplement: Figure 5—source data 1. — Values in parentheses are for the outer shell. ASU = asymmetric unit; R.m.s. = Root mean square. DOI: http://dx.doi.org/10.7554/eLife.20930.028 [file elife-20930-fig5-data1.docx]

|  | **Pcdh γA4_EC3–6_** | **Pcdh γB2_EC3–6_** |
| --- | --- | --- |
| **Data collection** |  |  |
| Date | 06/29/2016 | 06/29/2016 |
| Beamline | APS 24ID-C | APS 24ID-C |
| Wavelength (Å) | 0.97919 | 0.97919 |
| Space group | *P*2_1_2_1_2_1_ | *P*4_1_2_1_2 |
| *Cell dimensions* |  |  |
| a, b, c (Å) | 31.91, 63.79, 345.60 | 104.75, 104.75, 352.14 |
| α, β, γ (°) | 90, 90, 90 | 90, 90, 90 |
| Resolution (Å) | 172.16–2.56 (2.67–2.56) | 39.08–2.30 (2.34–2.30) |
| No. of reflections | 98304 | 485198 |
| Unique reflections | 23763 | 87920 |
| R_merge_ | 0.112 (3.118) | 0.119 (1.886) |
| CC(1/2) | 0.998 (0.434) | 0.998 (0.882) |
| I/σI | 6.6 (0.3) | 6.0 (0.8) |
| Completeness (%) | 99.3 (97.5) | 99.8 (99.9) |
| Redundancy | 4.1 (3.3) | 5.5 (5.6) |
| **Refinement** |  |  |
| Resolution (Å) | 20–3.0/4.5/2.6 | 20–2.3 |
| Unique reflections | 11653 | 86457 |
| Completeness in diffracting sphere/ ellipsoid* (%) | 99.0* | 98.3 |
| R_work_ / R_free_ (%) | 25.06 / 28.20 | 24.99 / 27.79 |
| Molecules in ASU | 1 | 3 |
| *Number of residues* |  |  |
| Protein | 422 | 1261 |
| Carbohydrate | 9 | 38 |
| Small molecule | 0 | 5 |
| Ion | 9 | 27 |
| Water | 0 | 276 |
| *R.m.s. deviations* |  |  |
| Bond lengths (Å) | 0.004 | 0.004 |
| Bond angles (°) | 0.658 | 0.650 |
| *Ramachandran* |  |  |
| Favored (%) | 95.67 | 97.44 |
| Allowed (%) | 4.33 | 2.56 |
| Outliers (%) | 0.00 | 0.00 |
| Rotamer outliers (%) | 2.75 | 2.64 |
| Wilson B | 48.68 | 45.36 |
| Overall B | 98.42 | 73.89 |
| Refined overall anisotropic scale matrix from Phenix | b_cart(11, 22, 33, 12, 13, 23): 13.4345, 243.7387, -21.6274, 0.0000, -0.0000, 0.0000 | V0: -2.4501, -4.2014, 4.4840, 0.5875, -1.3063, 0.6150  V1: -0.0042, -0.0064, 0.0361, -0.0047, 0.0018, -0.0008 |
| PDB ID | 5SZQ | 5SZR |

#### Figure 5—source data 1. X-ray crystallography data collection and refinement statistics for EC3–6 crystal structures

Values in parentheses are for the outer shell. ASU = asymmetric unit; R.m.s. = Root mean square.
